# A Call for Better Opioid Prescribing Training and Education

**DOI:** 10.5811/westjem.2016.8.31204

**Published:** 2016-10-03

**Authors:** Hazar Khidir, Scott G. Weiner

**Affiliations:** * Harvard Medical School, Boston, Massachusetts; † Harvard Medical School, Brigham and Women’s Hospital, Department of Emergency Medicine, Boston, Massachusetts

## Abstract

Pain is the most common complaint in the emergency department (ED), and emergency physicians face unique challenges in making opioid-related treatment decisions. Medical students and residents experience significant variation in the quality of education they receive both about opioid prescribing as well as substance-use detection and intervention in the ED. To achieve a better standard of education, clinical educators will need to (a) develop a clearer understanding of the risk for aberrant opioid prescribing in the ED, (b) recognize prescribing bias and promote uptake of evidence-based opioid prescribing guidelines in their EDs, and (c) advocate for integrated opioid management and addiction medicine training formally into medical school curricula.

## INTRODUCTION

At the start of training, medical students crave and cherish each patient encounter, are idealistic about the healing power of medicine, and have peak levels of empathy and benevolence.^1^ In their preclinical years, they are assessed by examinations that reinforce the principle that there is only one correct answer. Thus, many students enter the wards with the expectation that there is always a “correct answer” to handling difficult clinical situations. In the hospital, they begin to be challenged with complex clinical encounters that do not meet these expectations.^2^

The emergency department (ED), where the incidence of complex social situations is particularly high and providers are expected to make quick decisions with limited information, exposes students to many challenging patient interactions.^3^ These interactions are further complicated by the fact that the emergency physician (EP) is expected to understand the episodic ED presentation in isolation, outside of the longitudinal care given by their usual providers. A particularly difficult clinical dilemma that students are likely to face in the ED is how to assess and manage patient analgesia requests that have the potential to result in opioid misuse. After spending some years in the hospital, nearly all residents and attendings become acquainted with the “drug-seeking patient” archetype characterized by a patient presenting with pain or symptoms, with that patient intending to solicit a prescription that will be misused or abused. At relatively more advanced stages of training, residents and attendings have likely developed their own unique approaches to these patient encounters.

For medical students in their early clinical training, this type of patient encounter is new and frustratingly equivocal. Voicing suspicion regarding aberrant medication-seeking behavior based on a patient’s medical history, specific requests, or behaviors can be uncomfortable for students, as it can feel like patient profiling and contradict their perceived role as benevolent caretaker. Compounding this discomfort is the very appropriate, overwhelming fear of unnecessarily and wrongly prolonging patient suffering. Conversely, as students develop a more pragmatic impression of medicine and work on building clinical acumen, they recognize the existence of patient dishonesty as a part of opioid addiction and are concerned about the dangers of inappropriate opioid prescribing.

Students have not garnered enough wisdom to feel confident in their assessment of the “legitimacy” of patient analgesia requests, and thus rely on their clinical educators for guidance. However, students often experience greatly varying attending approaches to these patient encounters. EP prescribing behaviors vary along a spectrum, even within a single institution.^4^,^5^ On one end of the spectrum, clinicians demonstrate a “sufferer” outlook toward these patients: giving credence to patients’ subjective pain reports, placing decision-making emphasis on the concern about undertreating pain, and demonstrating a high propensity to prescribe opioids. On the other end of the spectrum, providers will demonstrate a “seeker” outlook: exhibiting mistrust toward patients’ self-reported pain and perhaps obtaining imaging to demonstrate lack of musculoskeletal pathology, placing decision-making emphasis on the risk for aberrant opioid use behavior and demonstrating a low propensity to prescribe opioids.

Medical students’ varying experiences are not just anecdotal. In a national survey, at least 10% of EPs indicated they were less likely and 10% indicated they were more likely to prescribe opioids when they were presented with identical case scenarios.^6^ Moreover, physicians were found to interpret patient behavior and statements like, “I need something strong” differently. Some physicians reported that they would be less likely to prescribe opiates after hearing this statement while others reported they would be more likely.^6^

Pain is subjective, but opioid prescribing decisions do not have to be altogether subjective and idiosyncratic. EPs have the potential to be role models and educators to the next generation of prescribers. While providing effective education on opioid prescribing is a responsibility that should be met by providers in all clinical settings, unique challenges make the ED a particularly difficult setting for making opioid prescribing decisions. In this paper, we advocate that EPs achieve a better standard of education on safe opioid prescribing by (a) developing a clearer understanding of the risk for aberrant opioid prescribing in the ED, (b) recognizing prescribing bias and promoting uptake of evidence-based opioid prescribing guidelines in their EDs, and (c) advocating for earlier integration of opioid management and addiction medicine training formally into medical school curricula.

## STRATEGIES FOR STRENGTHENING OPIOID TRAINING

### A. Developing a Clearer Understanding of the Risk of Aberrant Opioid Prescribing in the ED

The ED is a setting where prescriptions for short-term opioids are frequently provided. Pain has been found to be the most common patient complaint in the ED with two-thirds of all visits being related to pain.^7^ Following a Joint Commission mandate in 2000 that hospitals better monitor and treat pain, rates of prescribing have overall increased, including in the ED where they were found to have nearly doubled over the past decade.^8^,^9^ However, opioid prescribing rates in the ED have decreased in Veterans Affairs settings since 2011.^10^ A recent cross-sectional study of 19 EDs estimates that 11.9% of all patients and 17% of discharged patients receive opioid prescriptions.^5^

Opioid prescriptions offered in the ED tend to be aligned with short-term treatment goals. EPs most commonly prescribe immediate-release combinations and are significantly less likely to prescribe high doses or large quantities of opioids, which are more strongly associated with morbid outcomes such as overdoses.^5^ But what’s the risk for misuse with short-term, low-dose opioids? And what factors are associated with a higher risk of misuse of short-term opioids?

There are few studies available that evaluate opioid use behaviors among patients discharged with opioids from the ED. One study does demonstrate that a percentage of those who are prescribed opioids progress to more frequent use; among patients who presented with low back pain and were prescribed opioids on ED discharge, 46% were still using opioid analgesia three months post-discharge.^11^ In another study, 36% of patients who were discharged from the ED with an opioid prescription self-reported medication misuse (defined as either self-escalating dose, use of prescription for a reason beside pain, or obtaining additional prescription opioids without a prescription) at 30-day follow up.^12^ In this study, there were no significant differences between opioid misusers and non-misusers with regard to gender, level of pain reported in the ED, amount of analgesia received at discharge, or discharge diagnoses.^13^

As EPs face the challenge of balancing their professional and moral duty to alleviate pain with their efforts to minimize opportunities for abuse of prescription opioids, more prospective studies on the degree to which short-term, low-dose opioid prescriptions lead to addiction are needed. Moreover, prospective studies could help identify risk factors that lead to misuse of or addiction to short-term, low-dose opioid prescriptions.

Findings from prospective studies would help better inform ED providers about the actual risks of different prescribing patterns, potentially leading to more evidence-based prescribing habits. This more robust evidence should then be shared in medical school classrooms to equip students with a clearer understanding of the physician role in the opioid epidemic. Findings would also provide students with evidence-based opioid-misuse screening tools to incorporate into their clinical algorithms for pain management decisions.

### B. Recognizing Prescribing Biases and Promoting Uptake of Evidence-based Opioid Prescribing Guidelines in the Emergency Department

EPs should critically assess their individual prescribing patterns to limit individual biases and assess how well they reflect prescribing guidelines. There are multiple biases that impact ED prescribing patterns. ED opioid-prescribing decisions are known to vary by race: White patients who present to the ED are more likely to receive opioid analgesia than any other racial group.^13^ Moreover, EPs were found to misidentify men as more likely to engage in aberrant behavior than women and place too much emphasis on patients’ suspicious history or pain symptoms when clinical impressions were compared to objective criteria from state prescription drug monitoring programs (PDMPs).^14^

EPs must limit perpetuation of long-standing racial and gender-related biases in clinical treatment to the next generation of opioid prescribers. EPs should reduce implicit bias by adapting their algorithms to emerging clinical guidelines and using evidence-based tools, including PDMPs to assess the risk of opioid misuse. Though recent studies have shown that PDMPs have not altered the average number of controlled substances prescribed per patient, providers perceived that PDMPs influenced their prescription decisions and felt more confident in their treatment decision after using these resources. 14

There are many guidelines that have been produced for prescribing opioids for chronic pain, and many of these have been critically evaluated by authorities such as the Centers for Disease Control and Prevention, National Institute on Drug Abuse, and Substance Abuse and Mental Health Services Administration.^14^ Some individual states and EP professional organizations have developed guidelines to inform the clinical practice of opioid prescribing. 16,17 Emerging opioid prescription guidelines have not yet been universally adopted by providers. This is likely related to limited awareness of new guidelines, physician uncertainty about the value of these guidelines and time constraints in keeping up with newly emerging recommendations.^19^

Hospital administrators should use strategies such as incorporating reminders and decision algorithms into electronic decision support systems or academic detailing to integrate clinical guidelines into everyday practice.^19^ Evidence-based guidelines would reduce inter-provider variability in prescribing practices. Not only would this translate to patients receiving more uniform care experiences, it would help ensure that medical students and trainees receive more consistent, evidence-based training on safe opioid prescribing in the hospital setting. Moreover, by making a concerted effort to ensure that evidence-based guidelines are implemented in the ED, EPs would be setting an example and modeling necessary safer prescribing reform not just for medical students and residents, but also for other types of providers whose prescribing habits are likely contributing to the opioid epidemic.

### C. Introducing and Integrating Opioid Management and Addiction Medicine Training Formally into Medical School Curricula

Individual EPs have tremendous potential to improve quality of opioid prescribing education by role-modeling evidenced-based prescribing approaches in the ED. However, education on opioid management should begin early, before medical students enter the wards. Residents have expressed sentiments of under-preparedness in making opioid prescription decisions.^20^ These sentiments likely stem from inadequate training in medical school, as medical students experience significant variation in the quality of education they receive on drug abuse detection and intervention.^20^ As these students and doctors in training are the next generation of providers inheriting the opioid epidemic, it is critical that clinical educators meet their need for earlier and better training on appropriate opioid prescribing.

The State of Massachusetts is beginning to recognize the need to aim interventions for managing the opioid crises at the roots. Massachusetts was the first state to develop a governmental initiative to reform opioid education for medical students and physicians in training, working with medical schools within the state to provide recommendations for and establish a commitment to medical school curriculum changes that would educate and train medical students on safe prescribing of opioids.^21^ Massachusetts medical schools have welcomed the intervention and made reforms to their students’ educational aims with one university already developing an educational strategy that will teach students how to identify patients at high risk for opioid misuse, how to treat pain in patients identified to be at high-risk for misuse, and how to manage substance use disorder chronically.^22^ The educational strategy will be comprehensive and integrated throughout the four years of medical school with students receiving classroom teaching on the science of addiction. Students will also complete clinical training modules on how to discuss substance use with patients, assess patients’ pain and symptoms, and use guidelines to come up with treatment decisions that maximize benefits and minimize harm.^22^

Educational strategies such as these will provide a structured, more evidenced-based foundation for medical students to begin developing their approach to preventing, diagnosing, and treating addiction. By incorporating such education into the formal curriculum, medical schools would be ensuring, rather than leaving to chance, that all students receive consistent, high-quality training on opioid use disorder prevention and treatment. National medical education accrediting bodies should develop similar initiatives to catalyze opioid education reform. As opioid use is a national problem, it is critical that the standard of education on safer opioid prescribing be raised uniformly across the United States.
